# Supplementary material for: Modeling glioblastoma heterogeneity as a dynamic network of cell states
Source: Mol Syst Biol. 2021 Sep 16;17(9):e10105. doi: 10.15252/msb.202010105 (PMC8444284; doi:10.15252/msb.202010105)
Supplement: Supplementary file 6 — Source Data for Figure 5 [file MSB-17-e10105-s004.zip › Figure5A_sourcedata/GSEA_3017/nosigdb_stateF.GseaPreranked.1622795015644/DOROSZKO_U3013MG_CLUSTER_3.html]

Details for gene set DOROSZKO\_U3013MG\_CLUSTER\_3[GSEA]

|  || Dataset | state33017 |
| Phenotype | NoPhenotypeAvailable |
| Upregulated in class | na\_pos |
| GeneSet | DOROSZKO\_U3013MG\_CLUSTER\_3 |
| Enrichment Score (ES) | 0.4945291 |
| Normalized Enrichment Score (NES) | 2.7996264 |
| Nominal p-value | 0.0 |
| FDR q-value | 0.0 |
| FWER p-Value | 0.0 |
Table: GSEA Results Summary

  

Fig 1: Enrichment plot: DOROSZKO\_U3013MG\_CLUSTER\_3      
 Profile of the Running ES Score & Positions of GeneSet Members on the Rank Ordered List

  

| PROBE | GENE SYMBOL | GENE\_TITLE | RANK IN GENE LIST | RANK METRIC SCORE | RUNNING ES | CORE ENRICHMENT || 1 | GDF15 |  |  | 2 | 2.198 | 0.0640 | Yes |
| 2 | CLU |  |  | 5 | 1.852 | 0.1178 | Yes |
| 3 | LGALS3 |  |  | 16 | 1.261 | 0.1505 | Yes |
| 4 | TNFRSF21 |  |  | 36 | 1.016 | 0.1719 | Yes |
| 5 | IFITM3 |  |  | 38 | 0.987 | 0.2006 | Yes |
| 6 | SQSTM1 |  |  | 54 | 0.914 | 0.2207 | Yes |
| 7 | BST2 |  |  | 55 | 0.910 | 0.2476 | Yes |
| 8 | TSC22D4 |  |  | 64 | 0.855 | 0.2692 | Yes |
| 9 | GLUL |  |  | 69 | 0.837 | 0.2921 | Yes |
| 10 | CRYAB |  |  | 85 | 0.783 | 0.3084 | Yes |
| 11 | MAPT |  |  | 163 | 0.644 | 0.2923 | Yes |
| 12 | IGFBP7 |  |  | 164 | 0.643 | 0.3113 | Yes |
| 13 | SEMA3E |  |  | 165 | 0.636 | 0.3300 | Yes |
| 14 | ADGRG1 |  |  | 184 | 0.606 | 0.3397 | Yes |
| 15 | TIMP2 |  |  | 196 | 0.590 | 0.3521 | Yes |
| 16 | AL355974.2 |  |  | 207 | 0.584 | 0.3648 | Yes |
| 17 | BLVRB |  |  | 235 | 0.565 | 0.3692 | Yes |
| 18 | SELENOP |  |  | 256 | 0.548 | 0.3763 | Yes |
| 19 | FAM162A |  |  | 282 | 0.524 | 0.3803 | Yes |
| 20 | PLA2G16 |  |  | 284 | 0.523 | 0.3953 | Yes |
| 21 | GSTK1 |  |  | 312 | 0.503 | 0.3979 | Yes |
| 22 | NRP2 |  |  | 313 | 0.503 | 0.4127 | Yes |
| 23 | ISCU |  |  | 325 | 0.498 | 0.4224 | Yes |
| 24 | PSAT1 |  |  | 329 | 0.496 | 0.4357 | Yes |
| 25 | CYSTM1 |  |  | 358 | 0.484 | 0.4372 | Yes |
| 26 | ID2 |  |  | 366 | 0.483 | 0.4483 | Yes |
| 27 | RAP2B |  |  | 372 | 0.480 | 0.4602 | Yes |
| 28 | CTSL |  |  | 376 | 0.479 | 0.4729 | Yes |
| 29 | ADI1 |  |  | 415 | 0.464 | 0.4693 | Yes |
| 30 | TKT |  |  | 431 | 0.459 | 0.4760 | Yes |
| 31 | TTYH1 |  |  | 457 | 0.448 | 0.4778 | Yes |
| 32 | PPIC |  |  | 462 | 0.445 | 0.4892 | Yes |
| 33 | LGALS3BP |  |  | 528 | 0.424 | 0.4720 | Yes |
| 34 | FAM198B |  |  | 536 | 0.418 | 0.4811 | Yes |
| 35 | MATN2 |  |  | 552 | 0.414 | 0.4865 | Yes |
| 36 | PMP2 |  |  | 562 | 0.411 | 0.4945 | Yes |
| 37 | ALKBH7 |  |  | 606 | 0.396 | 0.4866 | No |
| 38 | NFE2L2 |  |  | 663 | 0.381 | 0.4723 | No |
| 39 | FABP7 |  |  | 701 | 0.371 | 0.4663 | No |
| 40 | PFN2 |  |  | 724 | 0.365 | 0.4671 | No |
| 41 | FSTL1 |  |  | 774 | 0.352 | 0.4551 | No |
| 42 | KCTD12 |  |  | 808 | 0.345 | 0.4502 | No |
| 43 | FCGRT |  |  | 814 | 0.344 | 0.4581 | No |
| 44 | IFI6 |  |  | 922 | 0.323 | 0.4187 | No |
| 45 | MDH1 |  |  | 924 | 0.322 | 0.4278 | No |
| 46 | EIF4B |  |  | 927 | 0.322 | 0.4364 | No |
| 47 | HES4 |  |  | 950 | 0.317 | 0.4357 | No |
| 48 | HIBADH |  |  | 979 | 0.313 | 0.4322 | No |
| 49 | REV3L |  |  | 999 | 0.310 | 0.4326 | No |
| 50 | CD9 |  |  | 1070 | 0.297 | 0.4094 | No |
| 51 | COX14 |  |  | 1163 | 0.283 | 0.3758 | No |
| 52 | CTNNA1 |  |  | 1228 | 0.276 | 0.3547 | No |
| 53 | VAT1 |  |  | 1237 | 0.275 | 0.3592 | No |
| 54 | CDH11 |  |  | 1240 | 0.275 | 0.3664 | No |
| 55 | CTSD |  |  | 1245 | 0.275 | 0.3727 | No |
| 56 | SNHG18 |  |  | 1314 | 0.267 | 0.3495 | No |
| 57 | ST8SIA1 |  |  | 1346 | 0.262 | 0.3431 | No |
| 58 | TEX264 |  |  | 1419 | 0.255 | 0.3177 | No |
| 59 | ACO2 |  |  | 1443 | 0.253 | 0.3147 | No |
| 60 | ITGB1BP1 |  |  | 1501 | -0.259 | 0.2963 | No |
| 61 | NSA2 |  |  | 1531 | -0.267 | 0.2910 | No |
| 62 | EMP1 |  |  | 1598 | -0.285 | 0.2692 | No |
| 63 | PITHD1 |  |  | 1623 | -0.292 | 0.2669 | No |
| 64 | PHLDA1 |  |  | 1718 | -0.324 | 0.2335 | No |
| 65 | VDAC2 |  |  | 1779 | -0.353 | 0.2165 | No |
Table: GSEA details [plain text format]

  

Fig 2: DOROSZKO\_U3013MG\_CLUSTER\_3: Random ES distribution      
 Gene set null distribution of ES for **DOROSZKO\_U3013MG\_CLUSTER\_3**
